# Mortality and reproducibility of calcium measurements in patients with hypercalcemia reporting to the emergency department of a tertiary German hospital

**DOI:** 10.1186/s12245-025-01052-6

**Published:** 2025-11-13

**Authors:** Franziska M. Himmels, Annika Krane, Thomas Osterholt, Christoph Hüser, Victor Suárez, Volker R. Burst, Matthias J. Hackl

**Affiliations:** 1 Emergency Department, Faculty of Medicine and University Hospital Cologne, University of Cologne, Cologne, Germany; 2 Department II of Internal Medicine and Center for Molecular Medicine Cologne (CMMC), Faculty of Medicine and University Hospital Cologne, University of Cologne, Cologne, Germany

**Keywords:** Hypercalcemia, Reproducibility, Mortality, Calcium measurement, Germany

## Abstract

**Background:**

Severe hypercalcemia often results in the referral of patients to the emergency department (ED), as life-threatening consequences are feared. However, the available literature concerning the causes of hypercalcemia, mortality and therapeutic responses in these patients is scarce.

**Methods:**

We retrospectively analyzed a cohort of 1310 patients with a total serum calcium concentration ≥ 2.65 mmol/l, who reported to the ED of the University Hospital Cologne, Germany, between January 1st, 2010, and March 31st, 2021, for any reason, investigating hypercalcemia-associated diagnoses, ECG changes, symptoms of hypercalcemia, the course of calcium values over the first 5 days and hospital mortality.

**Results:**

The most common causes of hypercalcemia were malignancies, primary hyperparathyroidism and dehydration. Patients with sarcoidosis and vitamin D intoxication had the highest mean calcium levels at presentation. In patients with mild hypercalcemia, elevated total calcium values were often not reproducible in consecutive samples. Hypercalcemia due to dehydration, sepsis and subsequent to cardiopulmonary resuscitation (CPR) resulted in lower mean calcium levels, which quickly normalized in the following days. Hypercalcemia was well controlled with the applied therapies, even in the majority of patients who died during their hospital stay. We found no major abnormalities in the ECG analysis, and no death due to cardiac arrhythmias was documented in the patient charts. The mortality rate of patients varied greatly depending on the cause of hypercalcemia. Patients with malignancies had high mortality irrespective of total calcium levels at admission, whereas patients with sarcoidosis and hyperparathyroidism had low mortality despite high calcium levels.

**Conclusion:**

We found no evidence for acute death due to hypercalcemia. The degree of hypercalcemia might not be the main factor influencing mortality in these patients. Given that mild hypercalcemia is often not reproducible in consecutive blood samples, persistent hypercalcemia should be confirmed before further work-up is initiated.

**Supplementary Information:**

The online version contains supplementary material available at 10.1186/s12245-025-01052-6.

## Background

Severe hypercalcemia leading to hypercalcemic crisis, defined by volume depletion, metabolic encephalopathy and gastrointestinal symptoms, is widely considered a life-threatening emergency [1]. However, since the first description of a hypercalcemic crisis due to hyperparathyroidism in 1938 [2], only several hundred cases have been reported in the literature. While hypercalcemia is a common metabolic perturbation that results in visits to the emergency department (ED), hypercalcemic crisis is a feared but rare endocrinologic emergency [3, 4].

To maintain stable extracellular calcium levels, uptake from the gut, excretion into the urine, storage in and release from the bone as a large calcium reservoir are tightly regulated. Interference with vitamin D metabolism (vitamin D intoxication, sarcoidosis, tuberculosis, and paraneoplastic vitamin D production), disturbed parathyroid hormone production (hyperparathyroidism and lithium intoxication) or bone erosion (neoplastic metastases) disturb these control mechanisms and lead to hypercalcemia. Hypercalcemia has also been reported in patients with dehydration, sepsis and subsequent to CPR, in which the underlying pathophysiology is less clear [5–7].

Changes in serum proteins result in altered total calcium levels, whereas biologically active ionized calcium can be unaffected. Hence, it is common to report total calcium values corrected for albumin or protein, most commonly using the correction formulas proposed by Payne et al. [8]. However, the usefulness of these formulas has been increasingly questioned [9–12]. Therefore, we analyzed only uncorrected total calcium values in our patient cohort, following a “choosing wisely” recommendation to do so [13].

By studying patients who reported for any reason to the ED of a tertiary hospital in Germany during a 10-year period and whose blood samples revealed elevated total calcium values, we wanted to investigate whether the degree of hypercalcemia or the underlying disease is more relevant for the prognosis of the patient.

## Methods

### Study design / ethical approval

This study was a single-center retrospective cohort study. The study was approved by the local ethics committee (24-1193-retro) and registered on clincialtrials.gov (NCT06467877).

### Setting

The study was conducted in the ED of the University Hospital of Cologne, a German tertiary university hospital. The inclusion period was January 1st, 2010, to March 31st, 2021. No follow-up data were collected.

### Participants

A database query in the clinical chemistry information system returned all patients, who received blood sampling in the ED. Patients with a total calcium concentration ≥ 2.65 mmol/l in the initial blood sample and who were at least 18 years old were included, irrespective of the reason they reported to the ED with during the inclusion period. These patients constituted the study population, no further exclusion criteria were applied. The patient selection process is shown in Suppl. Figure 1. Owing to the blinding of the extracted data, informed consent from the patients was waived.

### Data sources / measurement

For this study, we reviewed the laboratory results, the electronic documentation of the ED physician, ICD10 codes assigned by medical coders for patients who were admitted to the hospital and the discharge letter of patients, which died in the hospital. All data was obtained by a single data abstractor and entered into a standardized table. Causes of hypercalcemia and symptoms were assigned from a prespecified list. (see respective methods sections and tables). The data abstractor was formally instructed, trained and supervised during data acquisition. The methodology employed in this work is in compliance with current recommendations on the conduct of chart reviews and the STROBE guidelines [14, 15].

Total calcium was measured in a centralized clinical chemistry laboratory onsite by adding NM-BAPTA and EDTA using a Calcium Gen.1 (2010–2012) or Calcium Gen.2 test (2012–2021, both Roche Diagnostics). Creatinine measurements were performed using the Creatinin Plus Gen 2 (Roche Diagnostics) test. The measurements were conducted on a Roche Modular P system (2010–2011) or on a Cobas C702 system (2011–2021). No formula was used to correct total calcium values, and no computational method to predict missing values was employed.

### Study size

The study size resulted from a query to the clinical chemistry database, which contains the data of all blood samples drawn in our ED, with the inclusion criteria specified in the participants section. The number of missing values for consecutive analyzes can be deducted from Suppl. Figure 1, in which the patient number for each analysis is listed. For the correlation of calcium and creatinine values we excluded patients on acute and chronic hemodialysis. Since the study was exploratory and descriptive no sample size calculation was performed.

### Causes of hypercalcemia

Causes of hypercalcemia were defined as previously published in the literature (Table 1) [3, 6, 7, 16, 17]. The diagnoses of dehydration and sepsis needed to be explicitly stated in physician documentation in the ED. The presence of one hypercalcemia-associated diagnosis was sufficient to categorize a patient accordingly. Cases with more than one hypercalcemia-associated diagnosis were attributed if a second reviewer agreed on the leading cause; otherwise, they were classified as unknown.

**Table 1:** Causes of hypercalcemia

	Calcium ≥2.65 mmol/l		Calcium ≥3.00 mmol/l		Reproducible hypercalcemia		Death in the hospital	
	*n* = 1310		*n* = 219		*n* = 424		*n* = 129	
Cause of hypercalcemia	n=	%	n=	%	n=	%	n=	%
Unknown	743	56.7%	36	16.4%	141	33.3%	32	24.1%
Malignancy	374	28.5%	139	63.5%	212	50.0%	73	54.9%
Primary hyperparathyroidism	53	4.0%	14	6.4%	27	6.4%		
Dehydration	52	4.0%	3	1.4%	10	2.4%	3	2.3%
Sepsis	31	2.4%	5	2.3%	6	1.4%	7	5.3%
Sarcoidosis	17	1.3%	13	5.9%	14	3.3%	1	0.8%
Resuscitation	14	1.1%	1	0.5%	2	0.5%	13	9.8%
Tertiary hyperparathyroidism	10	0.8%			6	1.4%		
Vitamin D intoxication	9	0.7%	5	2.3%	5	1.2%		
Excessive calcium substitution	3	0.2%	2	0.9%				
Long lie trauma	2	0.2%						
Lithium-induced	1	0.1%						
Tuberculosis	1	0.1%	1	0.5%	1	0.2%		

### Reproducibility analysis

Patients were stratified according to their initial total calcium value. We analyzed patients with at least two measurements of total calcium within 5 days of admission. If two or more total calcium values were ≥ 2.65 mmol/l, hypercalcemia was considered reproducible.

### Mortality analysis

For inpatients, we analyzed discharge codes for death in the hospital. For patients who were transferred to our palliative care ward, which for billing reasons involved administrative discharge and immediate readmission, we reviewed the discharge letter from this ward if patients died during their stay there.

### Correlation of calcium and creatinine values

Patients with a calcium/creatinine pair at presentation to the ED and at least one pair from a consecutive 24-hour period (24–48 h, 48–72 h, 72–96 h, or 96–120 h) who did not receive acute or chronic dialysis treatment were included. This strategy was employed to avoid bias caused by multiple measurements in the initial treatment phase and dialysis-induced creatinine fluctuations.

### Symptoms and treatment of hypercalcemia

For patients with total calcium concentrations above 3.0 mmol/l, we performed an in-depth chart review regarding symptoms of hypercalcemia, ECG changes and applied hypercalcemia treatments. Patient-reported complaints documented by the ED physician were matched to previously published symptoms [3]. Multiple symptoms in a single patient were counted in each respective category. The applied treatments for hypercalcemia were derived from physician documentation in the ED. Combination therapies were regarded as separate groups.

### ECG analysis

A single data abstractor analyzed ECGs, which were recorded as part of the ED admission procedure, regarding PQ intervals, QRS durations and QTc times. Follow-up ECGs were selected as close to discharge as possible and analyzed with respect to the total calcium value at that time.

### Statistical analysis

**Fig. 1:**
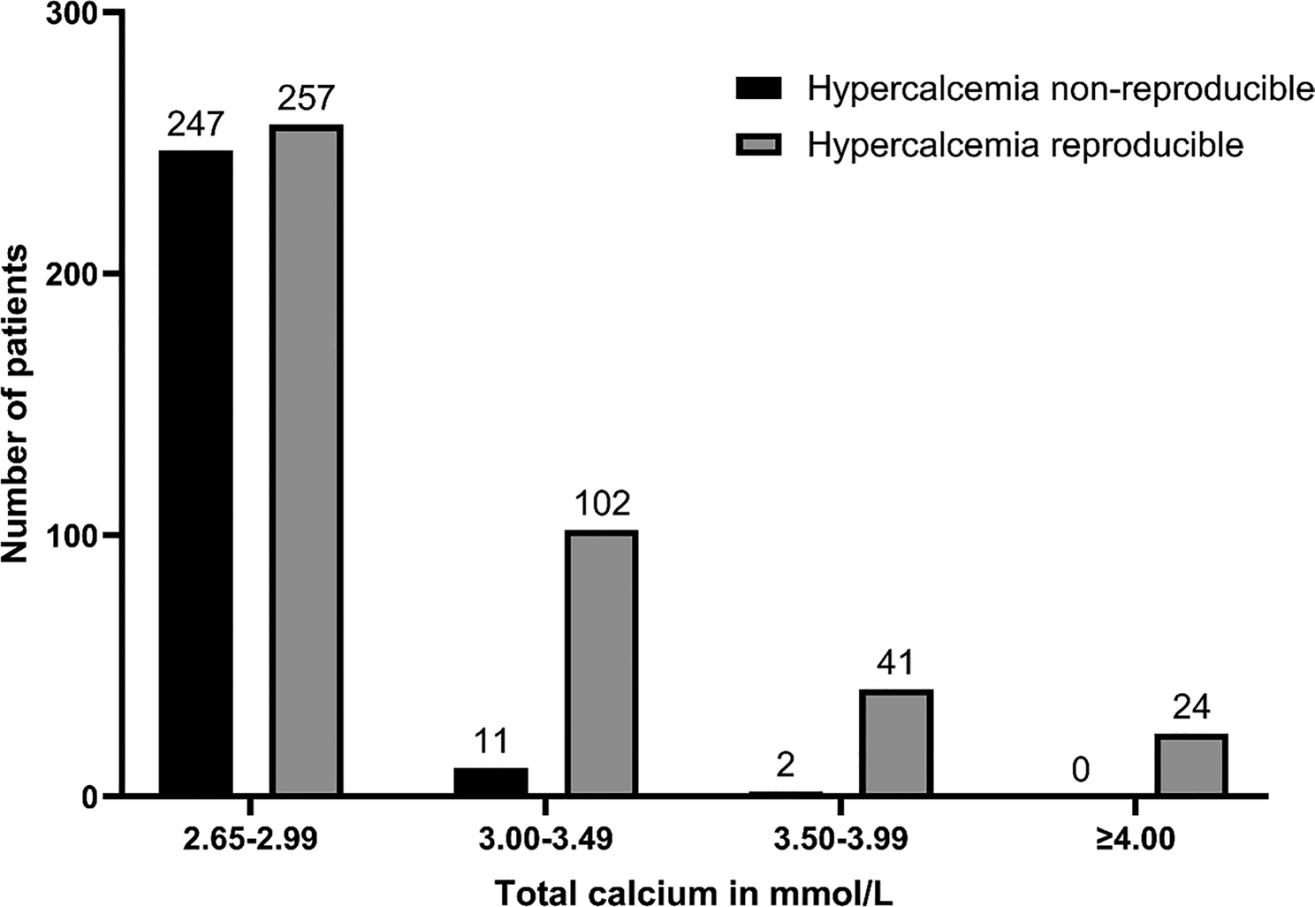
Number of patients with reproducible hypercalcemia. Patients with at least two measurements of total calcium within 5 days of admission were included in this analysis. Hypercalcemia was classified as reproducible if two or more of these measurements returned a total calcium concentration ≥ 2.65 mmol/l. The patients were grouped by their initial total calcium levels. *n* = 684

**Fig. 2:**
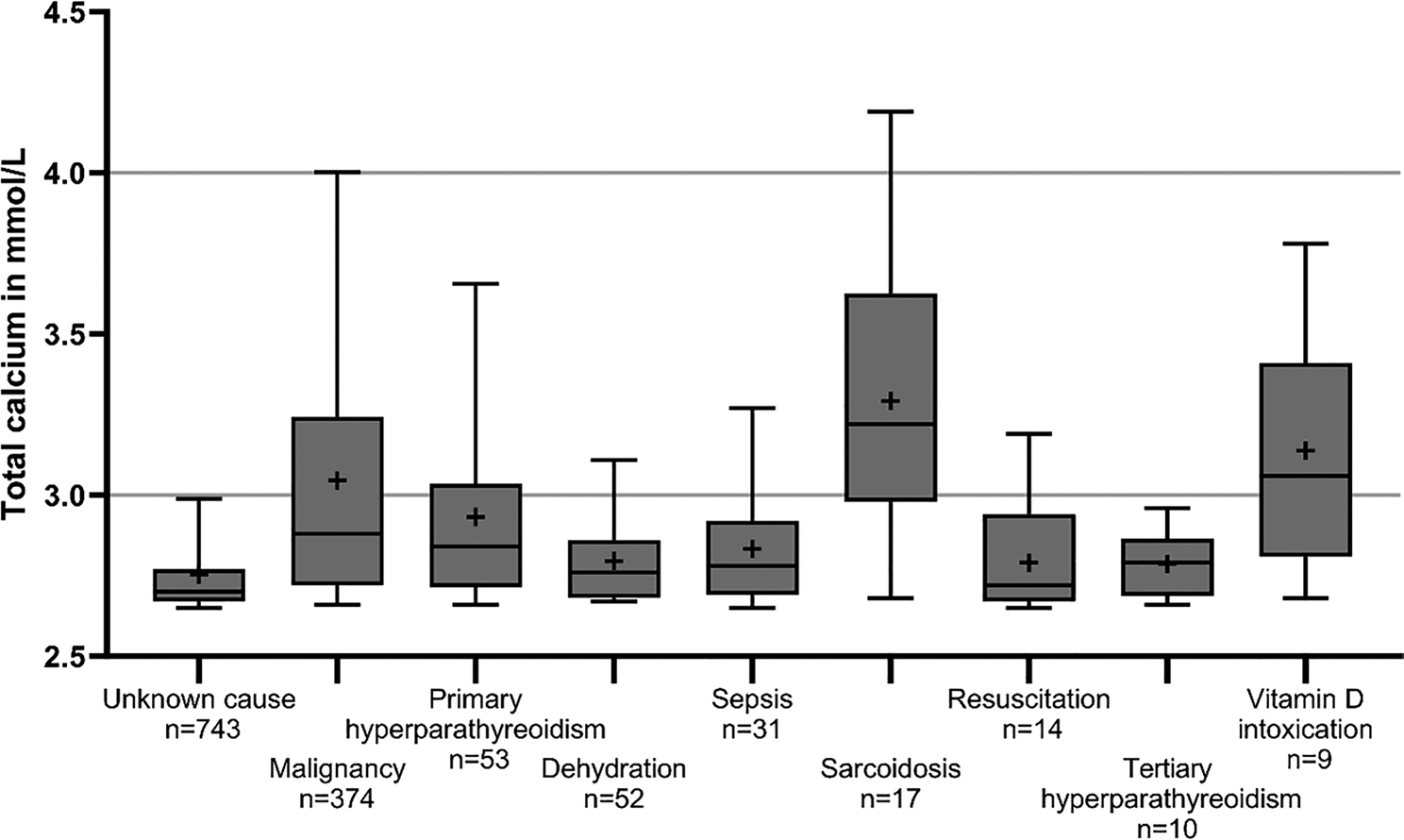
Total calcium at presentation to the ED. The total calcium values in the first blood sample in the ED are visualized as a box plot grouped by the cause of hypercalcemia. The first quartile, median and third quartile are depicted by the box; the minimum and maximum are depicted by whiskers; and the mean is depicted by +. Outliers are not shown

**Fig. 3:**
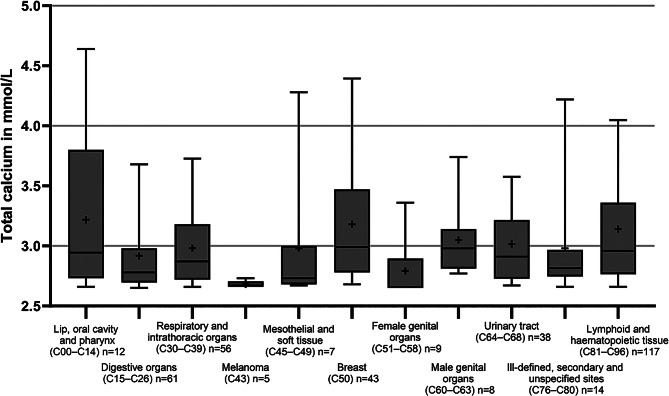
Total calcium at presentation to the ED in patients with hypercalcemia due to malignancies. Total calcium values in the first blood sample in the ED are visualized as a box plot grouped by the subtype of malignancy by ICD codes (Malignancies of the….). The first quartile, median and third quartile are depicted by the box; the minimum and maximum are depicted by whiskers; and the mean is depicted by +. Outliers are not shown

**Fig. 4:**
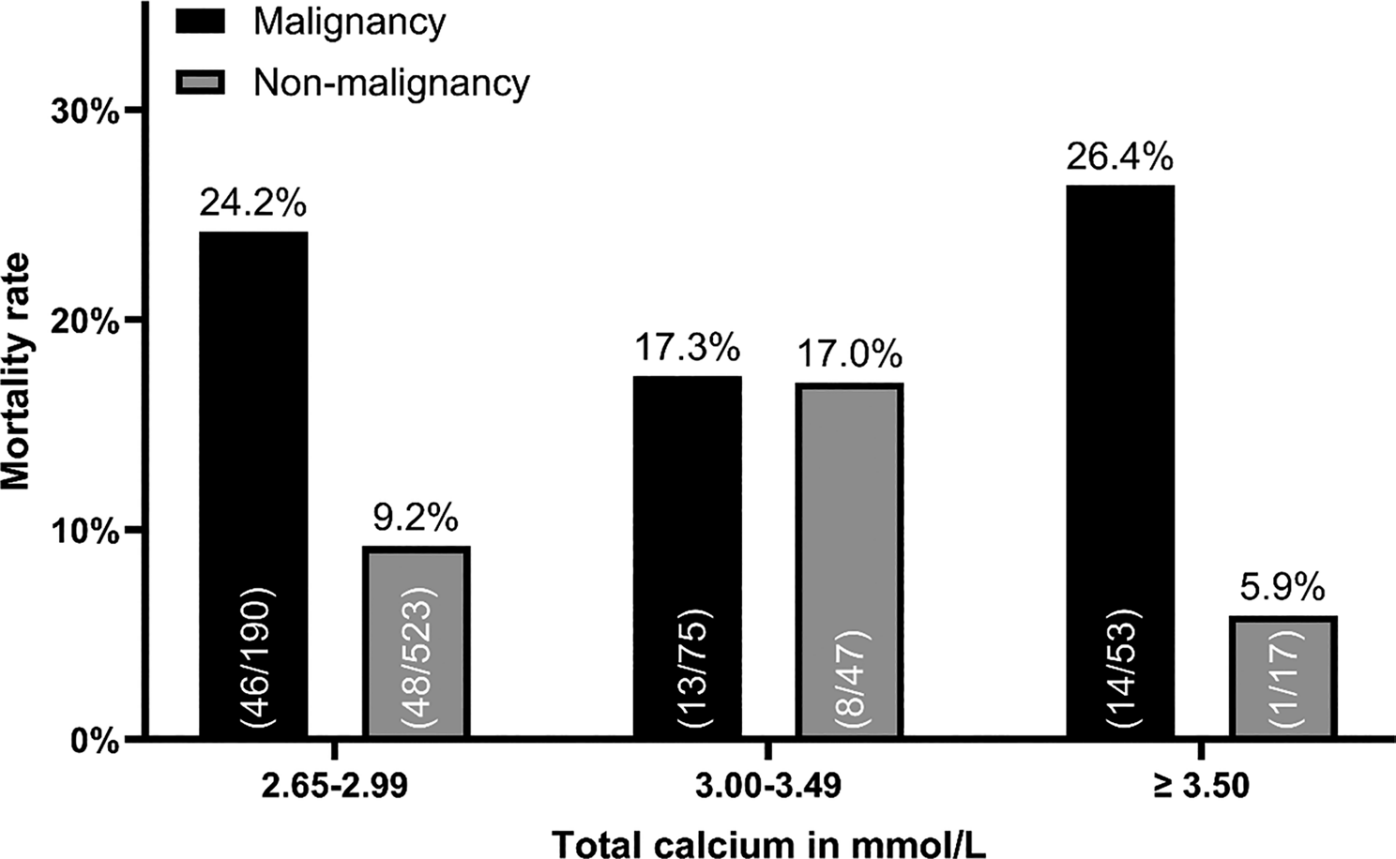
The mortality rate of patients depends on the initial total calcium level and the cause of hypercalcemia. Patients were stratified on the basis of their total calcium value at presentation to the ED. The percentage of patients who died is shown for patients with malignancies and for patients with any cause of hypercalcemia other than malignancies

**Fig. 5:**
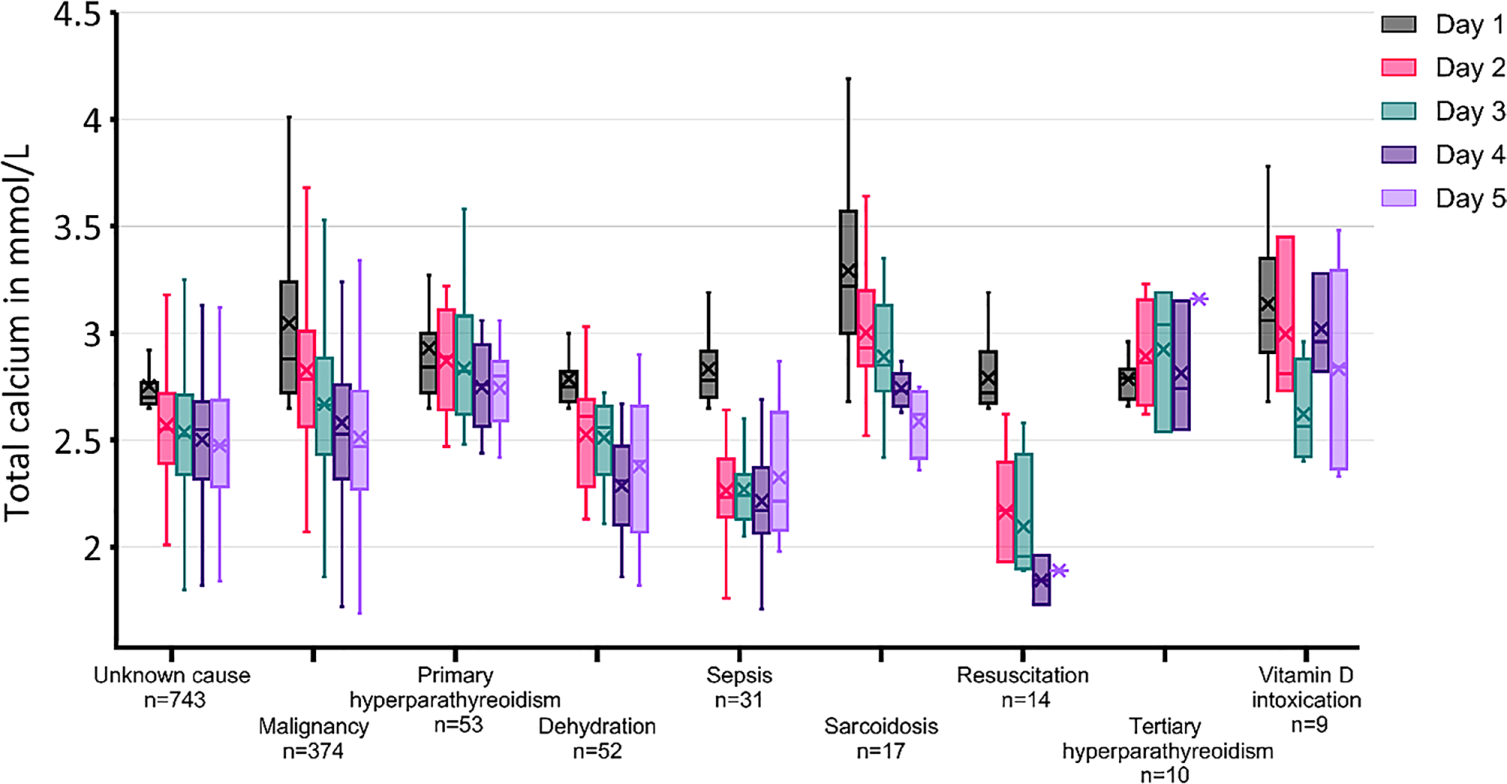
The course of total calcium values in the first 5 days differed depending on the cause of hypercalcemia. The total calcium values at presentation to the ED (day 1) and the first calcium value in each subsequent 24-hour period (24–47 h, 48–71 h, 72–95 h, etc.) are shown as box plots grouped by the cause of hypercalcemia. The first quartile, median and third quartile are depicted by the box; the minimum and maximum are depicted by whiskers; and the mean is depicted by +. Outliers are not shown. Causes of hypercalcemia in *n* < 5 patients are not shown

For the descriptive analysis, numerical variables are displayed as medians (interquartile ranges [IQRs]), and categorical and dichotomous variables are given as frequencies and proportions (%), respectively. Significance was calculated via two-tailed t tests for dependent or independent samples (stated in the text) after checking for normality and skewness. If the data was skewed, a Mann-Whitney-U test was performed for the comparison of independent groups and a Wilcoxon rank sum test was performed for the comparison of dependent groups. Analysis of the association between serum creatinine and calcium was performed by applying a repeated measures correlation [18] after checking for normality and skewness. Statistical analysis was performed using GraphPad Prism (version 10.6.1 (892)) and R (R Core Team, R version 4.2.0 (2022-04-22)). Due to the descriptive nature, no statistical analysis was performed in Figs. 1, 2, 3, 4 and 5 and Suppl. Figures 2, 3, 5 and 6.

## Results

During the period January 2010 to March 2021, 330,037 patients reported to the ED of the University Hospital Cologne. In 114,687 of these patients, a total serum calcium measurement was performed. In 1,310 of these blood samples, total calcium was elevated ≥ 2.65 mmol/l (1%). Of these, 669 (51%) were female, and the mean age was 60.4 ± 17.2 years. The mean total calcium at presentation to the ED was 2.86 ± 0.31 mmol/l.

For 580 patients (43%), a cause of hypercalcemia could be attributed by the available information in the ED physician’s letter and ICD discharge codes for inpatients (Table 1). The most common causes of hypercalcemia were malignancies (374; 29%), followed by primary hyperparathyroidism (53; 4%), dehydration/hypovolemia (52; 4%) and sepsis (31; 2%). The most common malignancies were hematological malignancies (117; 31%), digestive system tumors (61; 16%) and lung tumors (56; 15%) (Suppl. Table 1). In 743 (57%) of the patients, no cause of hypercalcemia could be identified in the available documentation. Stratification of patients according to the initial calcium value revealed that the vast majority of patients with unknown causes of hypercalcemia had mild hypercalcemia (total calcium values between 2.65 and 2.99 mmol/l, Suppl. Figure 2). As measurement fluctuations could also be responsible for these “unexplained” hypercalcemias, the reproducibility of elevated total calcium values was analyzed. In 49% of patients with a mild hypercalcemia, the calcium concentration was less than 2.65 mmol/l in all subsequent blood samples (Fig. 1). In the subgroup of patients with a calcium between 2.65 and 2.79 mmol/l the percentage of non-reproducible elevated calcium measurements was with 58% even higher (Suppl. Figure 3). Accordingly, the share of unknown causes of hypercalcemia decreased to 16% when using a total calcium cutoff of 3.00 mmol/l or 33% in the subgroup of patients with reproducible calcium measurements (Table 1).

The highest mean total calcium values were found in patients with hypercalcemia due to sarcoidosis and vitamin D intoxication, followed by those with solid tumors and primary hyperparathyroidism (Fig. 2). Patients without a hypercalcemia-associated diagnosis had the lowest mean total calcium values, followed by patients with hypercalcemia due to dehydration/hypovolemia, sepsis or subsequent to CPR. To further analyze hypercalcemia in patients with malignancies (374), tumor entities were grouped by ICD10 codes. The highest mean total calcium values were found in patients with breast cancer, cancer of the male reproductive system, hematologic malignancies, oropharyngeal tumors and cancer of the urinary tract. (Fig. 3)

Among the 905 inpatients, 129 died during the hospital stay, which resulted in a mortality rate of 14.3% for this cohort. Two groups were formed containing patients with hypercalcemia due to malignancy and patients with hypercalcemia from all other causes and then stratified by calcium values. The in-hospital mortality rate for patients with malignancies was 24.2% in patients with mild hypercalcemia (2.65–2.99 mmol/l), 17.3% in patients with moderate hypercalcemia (3.00–3.49 mmol/l), and 26.4% in patients with a total calcium level ≥ 3.50 mmol/l (Fig. 4). For patients with hypercalcemia due to other causes, the mortality rate rose from 9.2% (2.65–2.99 mmol/l) to 17.0% in patients with a total calcium concentration between 3.00 and 3.49 mmol/l. Remarkably, it was only 5.9% in patients with a total calcium level ≥ 3.50 mmol/l, in which sarcoidosis (6/17) and primary hyperparathyroidism (4/17) were the most common diagnoses (Fig. 4). Surprisingly, the total calcium values of patients who were admitted with calcium values ≥ 3.0 mmol/l and died during the hospital stay were well controlled within the first few days. The mean final available total calcium value before death was 2.61 ± 0.47 mmol/l (Suppl. Figure 7). In none of these patient charts were an unexpected sudden death or severe arrhythmias documented, suggesting that in the majority of these patients not the uncontrolled hypercalcemia was the reason of death.

To investigate the association between acute kidney injury (AKI) and hypercalcemia, first patients on acute or chronic hemodialysis were excluded, reducing the study population to 1176 patients. For 592 patients calcium and creatinine pairs at admission and during the following 5 days were available. Using a repeated measures correlation, the regression coefficient for calcium and creatinine was calculated as *r* = 0.19 (95% CI: 0.15–0.23), indicating a significant but weak correlation (Suppl. Figure 4a). While the mean calcium values declined remarkably, the mean creatinine values declined only slightly (Suppl. Figure 4b and 4c).

Patients with moderate/severe hypercalcemia (calcium ≥ 3.00 mmol/l, 219) were further analyzed regarding symptoms, ECG changes and hypercalcemia treatment. The most commonly reported symptom was deterioration of the general condition (125; 57%), followed by neuropsychiatric symptoms (39; 18%), gastrointestinal (GI) symptoms (48; 22%) and musculoskeletal symptoms (8; 4%). However, 12% of the patients (27) reported that they had noticed no symptoms despite the elevation of total calcium above 3.0 mmol/l. (Suppl. Table 2).

Next, the initial ECGs (151) were examined. The mean PQ interval, QRS width and QTc time were within the normal range [19] and did not significantly differ between patients with total calcium values between 3.00 - 3.99 mmol/l and ≥ 4.00 mmol/l or between two ECGs in a single patient (37) before and after calcium lowering treatment (Suppl. Table 3).

In 209 patients with moderate/severe hypercalcemia, who were not on maintenance dialysis, all calcium lowering treatments effectively reduced calcium in the first 5 days after admission. (Suppl. Figure 5). The analysis returned similar results when we included only patients with hypercalcemia due to malignancies (Suppl. Figure 6) but revealed differences when we sorted the patients by the cause of hypercalcemia. While hypercalcemia quickly resolved in patients with dehydration, sepsis and hypercalcemia subsequent to CPR, declined only slowly in patients with hyperparathyroidism and vitamin D intoxication despite treatment (Fig. 5).

## Discussion

We found no evidence for severe rhythm disorders or sudden cardiac deaths in the patient charts, and the ECG analysis revealed no differences, which is in line with the absence of life-threatening arrhythmias in patients with calcium levels above 4.0 mmol/l [20, 21] (Suppl. Table 3). This discrepancy to historical reports of acutely lethal hypercalcemias [1, 2] might be explained by the former widespread use of cardiac glycosides, which have been reported to potentiate arrhythmias in hypercalcemia [22].

The inpatient mortality rate differed markedly depending on the cause of hypercalcemia (Fig. 4). The mortality rate was lowest in the subgroup of patients without malignancies and with a calcium level ≥ 3.50 mmol/l, in which sarcoidosis and primary hyperparathyroidism were the most common diagnoses. In line with this, none of the patients with hyperparathyroidism and vitamin D intoxication and only one patient with sarcoidosis died, whereas 13 out of 14 patients (93%) with hypercalcemia following CPR, 23% of patients with sepsis and more than 20% of patients with malignancies died during the hospital stay. As demonstrated before [23], hypercalcemia was controllable within 3 days in patients with calcium levels ≥ 3.0 mmol/l who died during their hospital stay (Suppl. Figure 7). Taken together, this can be seen as a hint, that mortality is more influenced by the underlying disease than by the degree of hypercalcemia. Other factors like age, sex or comorbidities are also likely to influence mortality. Therefore, a multivariable regression analysis based on a larger patient population would be needed to answer this question.

All applied hypercalcemia therapies were similar in their effectiveness in controlling hypercalcemia (Suppl. Figures 5 and 6). However, it can be assumed that in the case of refractory hypercalcemia, the treatment was intensified until a sufficient treatment response was achieved. Hyperparathyroidism and vitamin D intoxication were least responsive to treatment in the first few days (Fig. 5), which could also be a result of a less aggressive treatment strategy.

The presented cohort with 1,310 patients is larger than comparable ED cohorts [3, 24, 25]. Similar to those cohorts, malignancies were the most common cause of hypercalcemia. Regional differences in the types of malignancies causing hypercalcemia most likely reflect varying prevalences of these tumors in the local patient population (Suppl. Table 1). The share of patients with hematologic malignancies was greater in this cohort than the share of hematologic malignancies among all cancer patients in Germany [26] (Suppl. Table 1). A similar overrepresentation of hematologic malignancies, as these are usually treated in specialized hematology centers, has also been reported in other cohorts, which also originate from tertiary hospitals [3, 16]. As in the other European cohorts, tuberculosis was a rare cause of hypercalcemia. Additionally, the data revealed, that hypercalcemia during hypovolemia, sepsis and subsequent to CPR was less severe (Fig. 2) and quickly normalized (Fig. 5), suggesting that a specific calcium-lowering therapy might often not be necessary in these patients.

For the majority of patients with slightly elevated total calcium values (2.65–2.80 mmol/l), hypercalcemia was not reproducible, and we found no attributable cause of hypercalcemia. The percentage of patients with nonattributable causes was lower in patients with calcium values above 3.00 mmol/l and in patients with reproducibly elevated calcium measurements (Table 1). Similar preselection strategies have been employed in other hypercalcemia cohorts [23, 24]. Therefore, it might be reasonable to repeat a calcium measurement in patients with slightly elevated calcium values before initiating further work-up for hypercalcemia.

Patients admitted to a hospital are at high risk of developing AKI during the hospital stay [27], and hypercalcemia has also been associated with an increased risk for AKI [28, 29]. Therefore, the relationship between calcium values and serum creatinine during treatment was investigated. This returned a weak correlation of declining calcium values with declining creatinine values, in contrast to the usual increase in creatinine in hospital patients after admission [27] (Suppl. Figure 4), likely due to hypercalcemia associated with dehydration [1].

The limitations of our study include its retrospective nature and the assumption that hypercalcemia is caused by a coexisting hypercalcemia-associated diagnosis. Identification of those diagnoses was limited to the available documentation, which might be incomplete. Especially in patients with only slight elevations in total calcium values, the coexistence of, e.g., a malignancy is not necessarily causative of a possibly nonreproducible total calcium elevation. As no follow-up data were available for discharged patients, the analyses were limited to data obtained during the hospital stay, and follow-up blood samples were available only at varying time points. The retrospective study precluded analysis of gradual treatment intensification, if calcium values did not decline as desired.

Taken together, our data suggest that the degree of hypercalcemia might not be the main factor influencing mortality, but further studies are warranted to answer this question. Measurements of slightly elevated total calcium values should be repeated before initiating a detailed work-up. Elevated calcium levels were well controlled by the available treatment options in the majority of patients.

## Supplementary Information

Below is the link to the electronic supplementary material.

Supplementary Material 1

## Data Availability

The datasets generated and analyzed during the current study are not publicly available for legal reasons but are available from the corresponding author upon reasonable request.
