# Supplementary material for: Mortality and reproducibility of calcium measurements in patients with hypercalcemia reporting to the emergency department of a tertiary German hospital
Source: Int J Emerg Med. 2025 Nov 13;18:239. doi: 10.1186/s12245-025-01052-6 (PMC12616977; doi:10.1186/s12245-025-01052-6)
Supplement: Supplementary file 1 — Supplementary Material 1 [file 12245_2025_1052_MOESM1_ESM.pdf]

Mortality and reproducibility of calcium measurements in patients with hypercalcemia reporting to the emergency department of a tertiary German hospital

International Journal of Emergency Medicine

Franziska M. Himmels, MD[1,2], Annika Krane, MD[1,2], Thomas Osterholt, MD[1,2], Christoph Hüser, MD[1,2], Victor Suárez, MD[1,2], Volker R. Burst, MD[1,2], Matthias J. Hackl, MD[1,2]

1) Emergency Department, University of Cologne, Faculty of Medicine and University Hospital Cologne, Cologne, Germany

2) Department II of Internal Medicine and Center for Molecular Medicine Cologne (CMMC), University of Cologne, Faculty of Medicine and University Hospital Cologne, Cologne, Germany

Corresponding author: Matthias J. Hackl
Email: matthias.hackl@uk-koeln.de

Supplemental Material

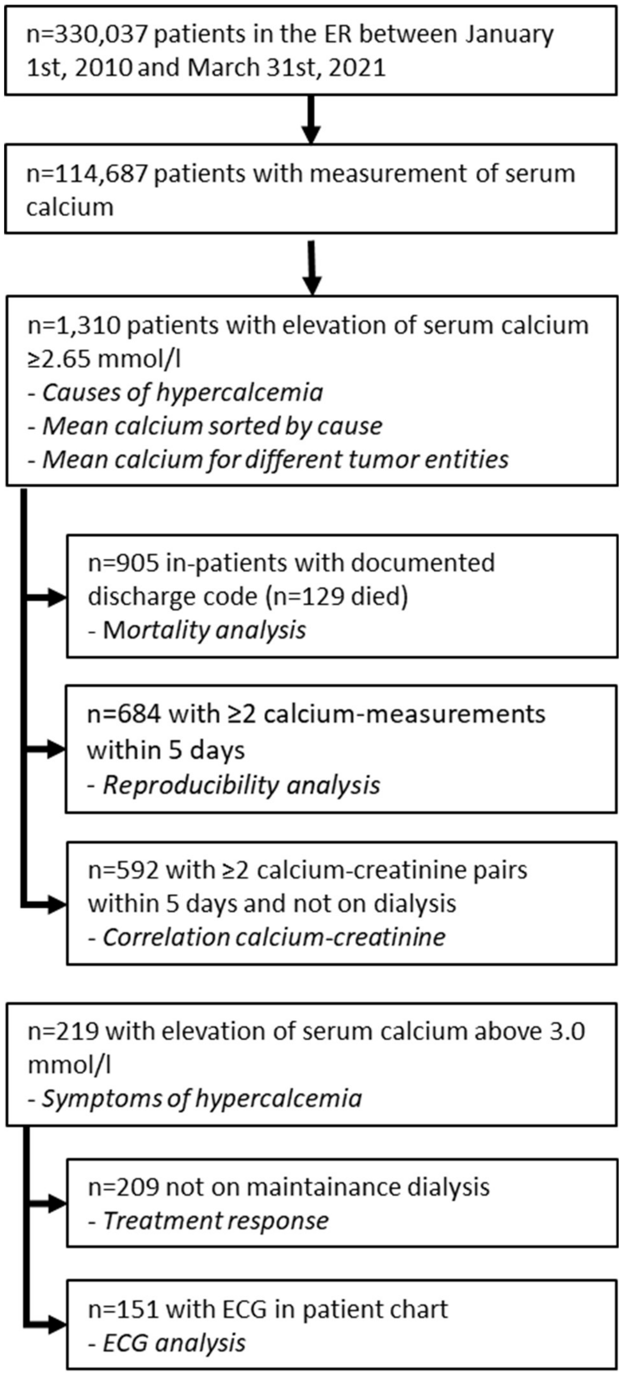

Suppl. Fig. 1 Flow diagram of patient selection

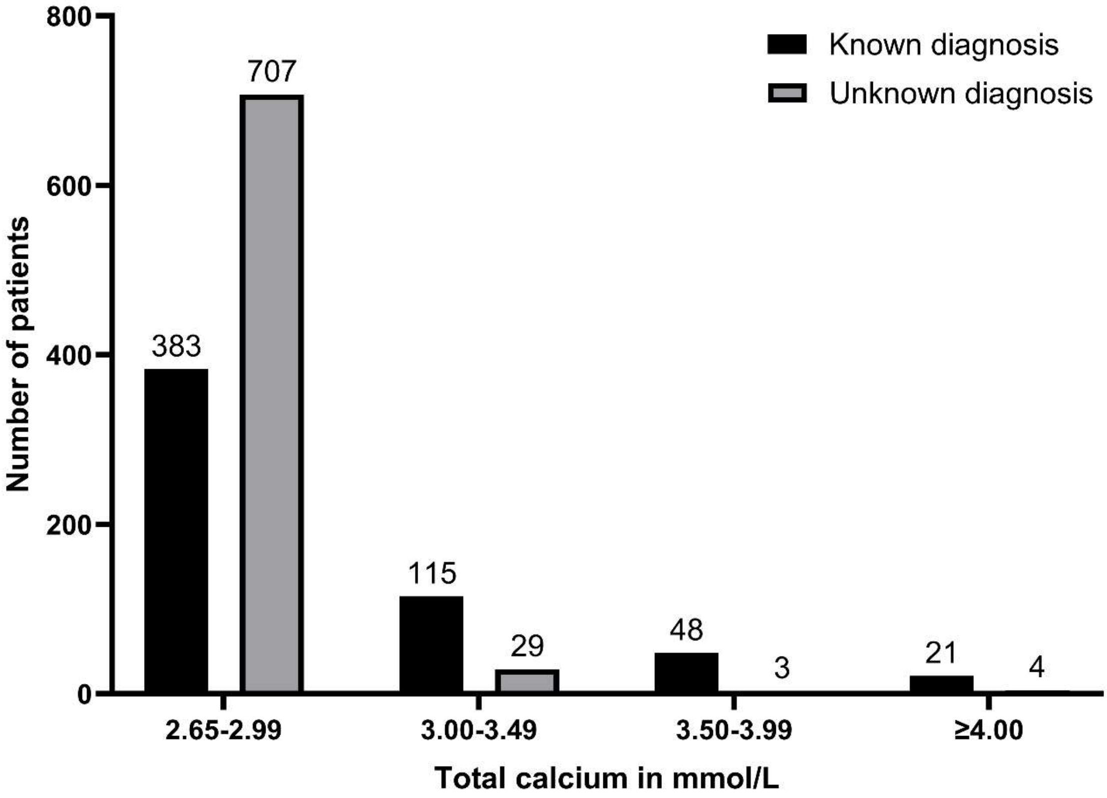

Suppl. Fig. 2 The share of patients with an unknown cause of hypercalcemia declines with increasing total calcium levels. While the majority of patients with total calcium levels between 2.65 and 2.99 mmol/l have an unknown cause of hypercalcemia, e.g. no hypercalcemia associated diagnosis could be found in the patient chart, this declines with increasing calcium levels to only a few patients in the group with the highest calcium values.

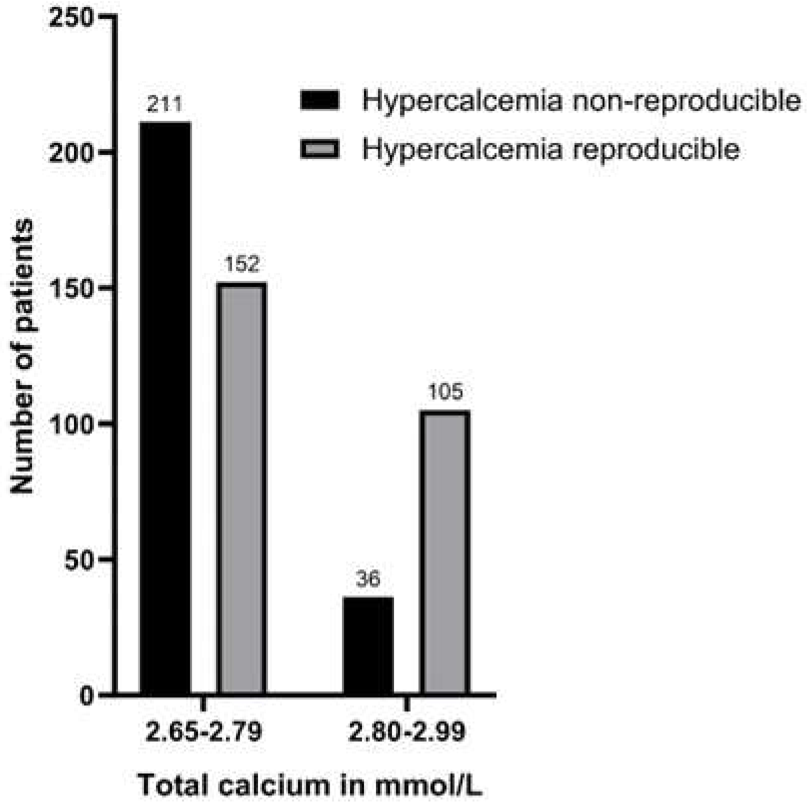

Suppl. Fig. 3 Slight elevations of serum calcium are often not reproducible. Included in this analysis were patients with at least two measurements of total calcium within 5 days of admission. Hypercalcemia was classified as reproducible if two or more of these measurements returned a total calcium ≥2.65 mmol/l. Patients with moderate hypercalcemia (2.65-2,99 mol/l) are shown subdivided into calcium elevations between 2.65-2.79 mmol/l and 2.80-2.99 mmol/l.

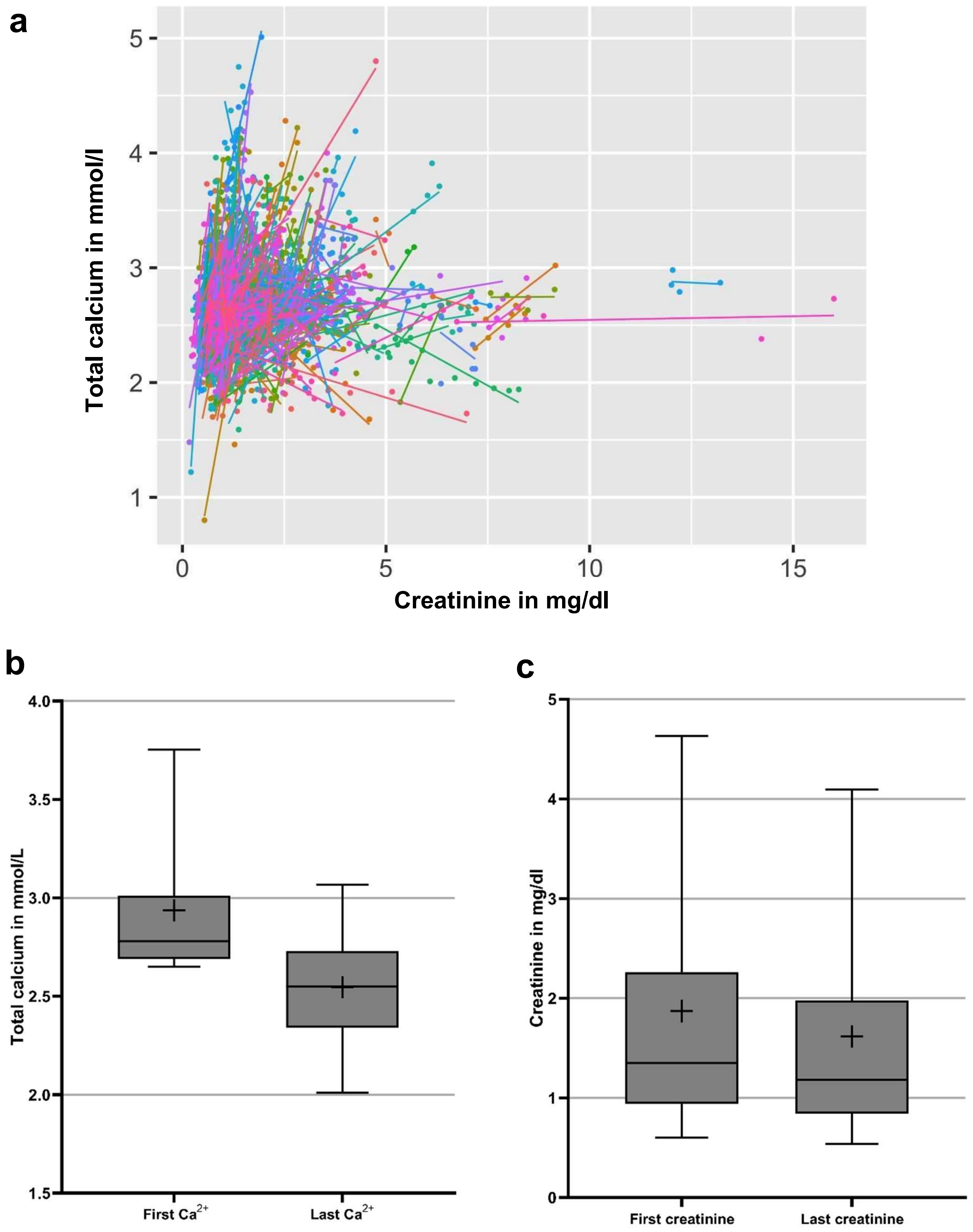

Suppl. Fig. 4 Total calcium and creatinine values show only a weak correlation in a single patient. Calcium/creatinine value pairs at presentation and within 5 days of admission were analysed regarding a correlation between calcium and creatinine. (**A**) The regression line of each patient is shown in the plot. As the measurements are not independent, we performed an analysis for repeated measurements (n=529). The resulting regression coefficient r = 0.19 (95% CI: 0.15 - 0.23, not shown) revealed only a very weak association. The first and last total calcium value (B) and creatinine value (C) are shown as box plots and demonstrate a decline of mean calcium and creatinine values over time.

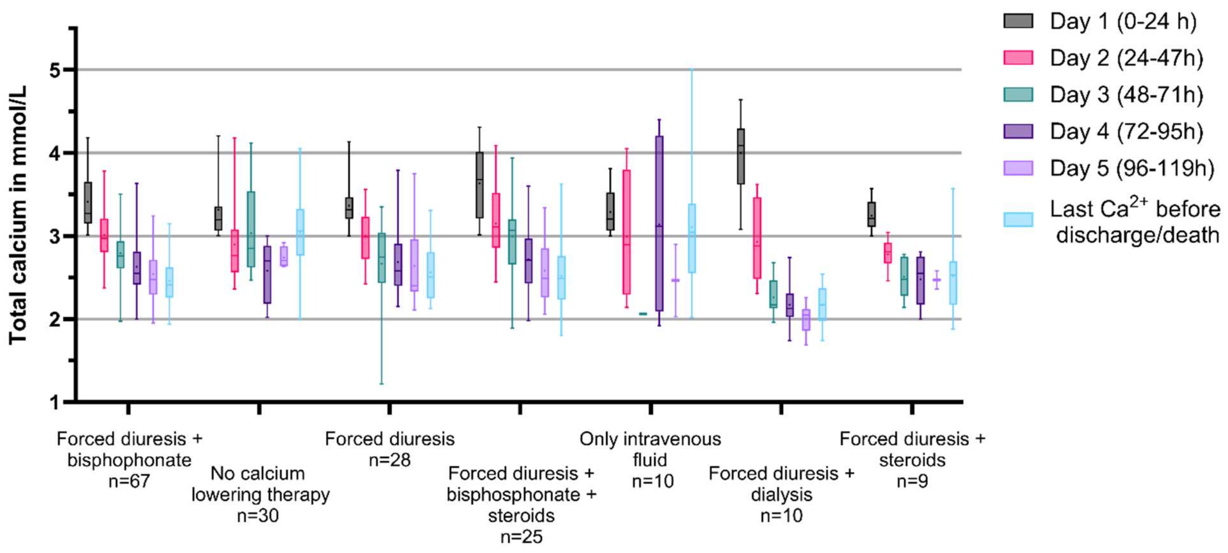

Suppl. Fig. 5 Treatment response in the first 5 days in patients with a total calcium ≥3.0 mmol/l. Shown are the calcium values at presentation to the ED (Day 1), the first calcium value in each following 24 hour period (24-47h, 48-71h, 72-95h, etc.) and the last available total calcium value before discharge or death as box plots grouped by the applied calcium lowering therapy. The first quartile, median and third quartile is depicted by the box, the minimum and maximum by whiskers and the mean by +. Outliers are not shown. Treatment regimens received by n≤5 patients are not shown. n=209 patients

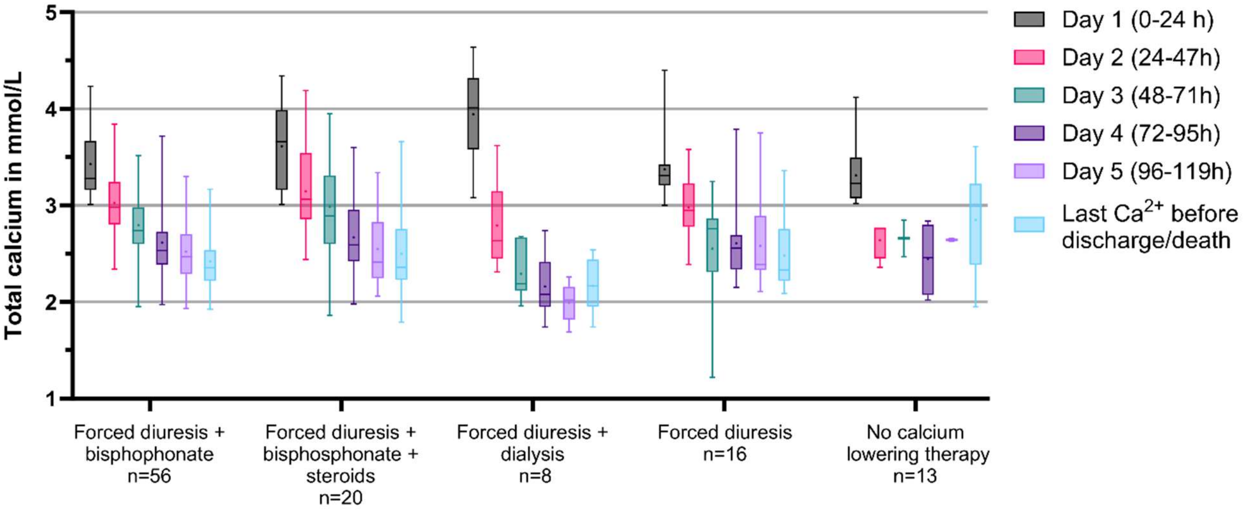

Suppl. Fig. 6 Treatment response in patients with a total calcium ≥3.0 mmol/l due to malignancies. Shown are the calcium values at presentation to the ED (Day 1), the first calcium value in each following 24 hour period (24-47h, 48-71h, 72-95h, etc.) and the last available total calcium value before discharge or death are depicted as a boxplot grouped by the applied calcium lowering therapy. The first quartile, median and third quartile is depicted by the box, the minimum and maximum by whiskers and the mean by +. Outliers are not shown. Treatment regimens received by n≤5 patients are not shown. n=136 patients

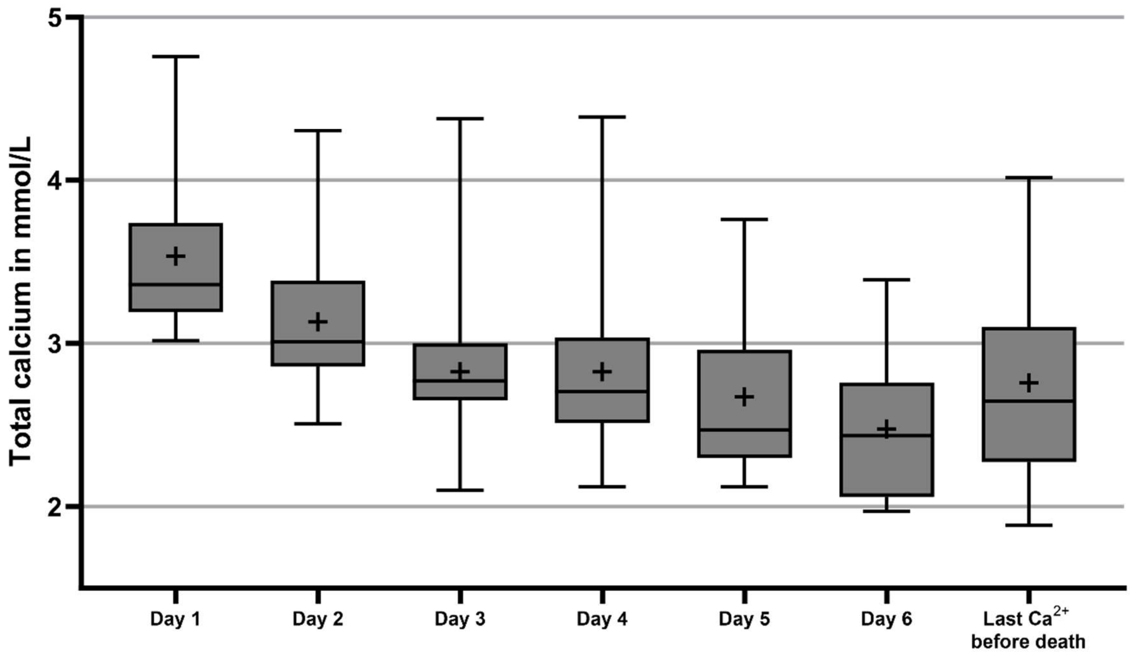

Suppl. Fig. 7 Hypercalcemia was well controlled in patients with an initial calcium ≥3.0 mmol/l who died during the hospital stay. Total calcium values at presentation to the ED (day 1), the first calcium value on each following day (24-47h, 48-71h, 72-95h, etc. after presentation to the ED) and the last available total calcium value before death are depicted as a boxplot. The first quartile, median and third quartile is depicted by the box, the minimum and maximum by whiskers and the mean by +. Outliers are not shown. n=36 patients

Cause of hypercalcemia	Calcium >= 2.65 n=	n=1310 %	Calcium >= 3.00 n=	n=219 %	Reproducible n=	n=424 %	Death n=	n=129 %
Unknown	743	56,7%	36	16,4%	141	33,3%	32	24,1%
Malignancy	374	28,5%	139	63,5%	212	50,0%	73	54,9%
Primary hyperparathyroidism	53	4,0%	14	6,4%	27	6,4%		
Dehydration	52	4,0%	3	1,4%	10	2,4%	3	2,3%
Sepsis	31	2,4%	5	2,3%	6	1,4%	7	5,3%
Sarkoidosis	17	1,3%	13	5,9%	14	3,3%	1	0,8%
Resuscitation	14	1,1%	1	0,5%	2	0,5%	13	9,8%
Tertiary hyperparathyroidism	10	0,8%			6	1,4%		
Vitamin D intoxication	9	0,7%	5	2,3%	5	1,2%		
Excessive ca substitution	3	0,2%	2	0,9%				
Long lie trauma	2	0,2%						
Lithium-induced	1	0,1%						
Tuberculosis	1	0,1%	1	0,5%	1	0,2%		

Suppl. Table 1 Hypercalcemia due to malignancies in comparison with published cohorts and the German cancer registry

**Symptoms of hypercalcemia**	n=	%
**General deterioration/Fatigue**		
Deterioration of general condition	107	49%
Fatigue	12	5%
Orthostatic disregulation	6	3%
**Neuropsychiatric symptoms**		
Disorientation	11	5%
Somnolence	28	13%
**Gastrointestinal symptoms**		
Diarrhea	2	1%
Obstipation	13	6%
Nausea	33	15%
**Muskuloskeletal symtpoms**		
Myopathy	8	4%
**Asymptomatic**	27	12%
**No information**	34	16%

Suppl. Table 2 Symptoms of hypercalcemia are unspecific

	Calcium 3.00-3.99 mmol/l n=131	Calcium ≥4.00 mmol/l n=20	p values	First ECG at presentation n=37	Second ECG during treatment n=37	p values
PQ in ms	155 ± 34	152 ± 21	0.526	150 ± 27	144 ± 22	0.287
QRS in ms	95 ± 22	98 ± 19	0.243	95 ± 23	90 ± 12	0.135
QTc in ms	427 ± 45	408 ± 48	0.117	416 ± 42	420 ± 40	0.690
Calcium in mmol/l	3.32 ± 0.27	4.26 ± 0.24		3.53 ± 0.54	2.75 ± 0.58	<0.001

Suppl. Table 3 Hypercalcemia does not result in major ECG changes

Times are listed as mean ± standard deviation. P values are shown for a t-test for independent samples (calcium at presentation) and a t-test for dependent samples (two ECGs in one patient). The data for QRS duration was skewed. Therefore, a Mann-Whitney-U test was performed for the comparison between the calcium levels on admission and a Wilcoxon rank sum test was performed for the comparison between first and second ECG. None of the p values for the ECG times reached statistical significance.
